# Dual R108K and G189D Mutations in the NS1 Protein of A/H1N1 Influenza Virus Counteract Host Innate Immune Responses

**DOI:** 10.3390/v13050905

**Published:** 2021-05-13

**Authors:** Meng-Ting Huang, Sen Zhang, Ya-Nan Wu, Wei Li, Yu-Chang Li, Chang-Shuai Zhou, Xiao-Ping Kang, Tao Jiang

**Affiliations:** 1 School of Basic Medical Sciences, Anhui Medical University, Hefei 230032, China; m18974010466@163.com; 2 State Key Laboratory of Pathogen and Biosecurity, Beijing Institute of Microbiology and Epidemiology, Academy of Milltary Medical Sciences, Beijing 100071, China; zhangsen2260@163.com (S.Z.); wyn1777@163.com (Y.-N.W.); 18567625401@163.com (W.L.); liyuchang66@163.com (Y.-C.L.); ZCS56017@163.com (C.-S.Z.); kangxiaoping@163.com (X.-P.K.)

**Keywords:** influenza A virus, NS1 protein, mutation, immune escape

## Abstract

Influenza A viruses (IAV) modulate host antiviral responses to promote growth and pathogenicity. Here, we examined the multifunctional IAV nonstructural protein 1 (NS1) of influenza A virus to better understand factors that contribute to viral replication efficiency or pathogenicity. In 2009, a pandemic H1N1 IAV (A/California/07/2009 pH1N1) emerged in the human population from swine. Seasonal variants of this virus are still circulating in humans. Here, we compared the sequence of a seasonal variant of this H1N1 influenza virus (A/Urumqi/XJ49/2018(H1N1), first isolated in 2018) with the pandemic strain A/California/07/2009. The 2018 virus harbored amino acid mutations (I123V and N205S) in important functional sites; however, 108R and 189G were highly conserved between A/California/07/2009 and the 2018 variant. To better understand interactions between influenza viruses and the human innate immune system, we generated and rescued seasonal 2009 H1N1 IAV mutants expressing an NS1 protein harboring a dual mutation (R108K/G189D) at these conserved residues and then analyzed its biological characteristics. We found that the mutated NS1 protein exhibited systematic and selective inhibition of cytokine responses via a mechanism that may not involve binding to cleavage and polyadenylation specificity factor 30 (CPSF30). These results highlight the complexity underlying host–influenza NS1 protein interactions.

## 1. Introduction

Influenza is an acute respiratory infectious disease caused by influenza virus infection. Influenza viruses belong to the family Orthomyxoviridae; there are four main types (influenza A, influenza B, influenza C, and influenza D) [1]. Influenza A and B viruses, which are the most common causes of seasonal and pandemic influenza in humans, have always been a major threat to human health [2]. IAV harbors a genome comprising eight single-stranded negative-sense RNA segments that encode at least 14 proteins [3]. Based on the properties of the hemagglutinin (HA) and neuraminidase (NA) surface proteins, IAVs can be divided into 18 different HA subtypes (H1–H18) and 11 different NA subtypes (N1–N11) [4].

The smallest nonstructural protein (NS) segment encodes two kinds of viral proteins (NS1 and NS2/NEP). The length of NS1 protein varies from 215–237 amino acids, depending on the strain of virus [5]. NS1 can be divided into two functional domains: the N-terminal RNA binding domain (RBD) and the C-terminal effect domain (ED), which interact with a variety of host factors. The RBD rotates to form a six-helix homodimer that binds to double-stranded RNA (dsRNA). The ED of most NS1 proteins contains nuclear output signals [6]. Therefore, the NS1 protein plays important roles not only in the cytoplasm but also in the nucleus [5]. NS1 is important for multiple viral functions, including temporal adjustment of viral RNA synthesis, control of viral mRNA splicing, adjustment of virus particle morphogenesis, and suppression of host apoptotic responses through activation of phosphoinositide 3-kinase (PI3K) [7]. The NS1 protein plays a crucial role in regulating host antiviral immune responses through various mechanisms. For example, NS1 increases virulence and/or pathogenicity in the host by acting as an interferon (IFN) antagonist, thereby enabling the virus to escape from interferon-mediated responses and replicate efficiently [8]. The RBD binds to both single-stranded RNA (ssRNA) and dsRNA, thereby blocking their recognition by retinoic acid-induced gene RIG-I and inhibiting expression of antiviral cytokines interferon-α/β [9]. NS1 also inhibits the 3′ terminal processing of host genes by binding to CPSF30 to block processing of cellular mRNAs [10].

However, the NS1 protein of the 2009 pandemic H1N1 IAV (pH1N1) virus lost the ability to bind CPSF30, so it cannot block expression of host genes, although studies show that this ability can be restored by introducing amino acid changes R108K, E125D, and G189D [11,12,13]. Interestingly, recombinant pH1N1 with these alternative expression NS1 can inhibit expression of host genes and is more effective than the 2009 pandemic H1N1 IAV (pH1N1) in combating host innate immune responses [14]. However, in vivo studies show that the NS1 mutant pH1N1 virus grows to a lower titer in the lungs of mice and has slightly lower pathogenicity than the WT virus [12,15]. There are vaccines against influenza viruses; however, these viruses mutate constantly, leading to increased virulence via mutation of viral proteins such as NS1. Since 2009, multiple amino acid sense mutations in the NS1 protein of pandemic H1N1 influenza virus have been reported, including the I123V and N205S mutations [12].

Here, we sequenced a seasonal 2009 H1N1 influenza virus isolated in 2018 and confirmed the presence of mutations I123V and N205S in important functional sites. However, 108R and 189G were conserved when compared with the early pandemic strain A/California/07/2009. To increase our understanding of the antagonistic mechanisms underlying interactions between influenza virus and host innate immunity, we used a reverse genetics approach to introduce a double mutation (R108K/G189D) into the NS1 protein of the seasonal 2009 H1N1 IAV, rescued the mutant virus, and analyzed its biological characteristics. We found that the mutated NS1 protein harboring R108K/G189D exhibited systematic and selective inhibition of cytokine responses beyond the complexity of host–influenza NS1 protein interactions.

## 2. Materials and Methods

### 2.1. Cell Culture and Virus

MDCK (Madin–Darby canine kidney) cells (ATCC, CCL-34), HEK293T (Human embryonic kidney) cells (ATCC, CRL-3216), and A549 (Human lung epithelial carcinoma) cells (ATCC, CCL-185) were cultured at 37 °C/5% CO_2_ in Dulbecco’s modified Eagle’s medium (DMEM, ThermoFisher, New York, NY, USA; 11965092) supplemented with 10% heat-inactivated fetal bovine serum (Gibco) and 1% PSG (100 U/mL penicillin, 100 μg/mL streptomycin). IAV A/Urumqi/XJ49/2018 (H1N1) (XJ49) was isolated from throat swabs obtained from patients in Urumqi in 2018 (GenBank Accession no.: MN549338–MN549345).

### 2.2. Sequence Analysis

According to their lineage/subtype, as well as the year, 2155 seasonally circulating NS1 sequences were downloaded from the influenza virus database of National Center for Biotechnology Information randomly (NCBI, https://www.ncbi.nlm.nih.gov/FLU, accessed on 10 November 2020). We used Molecular Evolutionary Genetic Analysis (MEGA) software v6.0 and Weblogo (http://weblogo.threeplusone.com/, accessed on 15 March 2020) for sequence alignment and mutation site analysis.

### 2.3. Generation of Recombinant Viruses by Reverse Genetics

The pHW2000 plasmid was kindly provided by Professor Robert G. Webster (Department of Pathology, University of Tennessee). The rXJ49 H1N1 virus was generated using an eight-plasmid system based on pHW2000 by transfection into human HEK293T cells. To obtain pHW2000 plasmids containing eight segments of IAV XJ49, viral RNA was extracted from infected cells and cDNA synthesized by reverse transcription using Uni12 primers [16]. The cDNA encoding the XJ49 viral genome was amplified using segment-specific primers and then cloned into the pHW2000 using BsmB I restriction enzymes, as described previously [16]. The R108K and G189D mutations were inserted into the NS1 gene using the Q5 site-directed mutagenesis kit (NEB; E0554S) and the following primers: NS1-R108K_F: 5’-CTTATGCCTAaGCAAAAGATAATAG-3’; NS1-R108K_R: 5’-CATGAACCAGTCTCGTGAC-3’; NS1-G189D_F: 5’-AAGTGGAATGaTAACACGGTTC-3’; and NS1-G189D_R: 5’-AAGTCCTCCGATGAGGAC-3’). The plasmid encoding the NS1 protein of XJ49 was substituted with the mutated NS1 plasmid to obtain the rXJ49-NS1mut virus. XJ49 and rXJ49-NS1mut were passaged in MDCK cells. Correct insertion of the viral segments into pHW2000 and presence of the R108K and G189D mutations were confirmed by sequencing.

### 2.4. Virus Growth Kinetics

To access virus growth kinetics in vitro, triplicate wells containing confluent monolayers of MDCK cells (5 × 105 cells/well, 6-well plates) were infected with viruses at a MOI of 0.01. After 1 h of virus adsorption at 37 °C, cells were overlaid with DMEM containing TPCK-treated trypsin (2 μg/mL) and then incubated at 37 °C. At 0, 12, 24, 48, 72, and 96 h post-infection (hpi), 500 μL cell supernatant was collected from each well, and the viral titer was determined in a plaque assay.

### 2.5. Plaque Assay

Confluent monolayers of MDCK cells (105 cells/well in a 12-well plate) were infected with virus at 37 °C. After 1 h of virus adsorption, cells were overlaid with DMEM containing 1% Low Melting Point agarose and TPCK-treated trypsin (2 μg/mL) and then incubated at 37 °C for 3 days. At 3 dpi, cells were fixed for 15 min at room temperature with 4% paraformaldehyde. The agarose overlay was removed and stained for 10 min with 1% crystal violet in methanol. The number of plaques in each well was counted.

### 2.6. Western Blot Analysis

Samples of transfected cells were prepared in buffer containing 100 mM Tris-HCl (pH 6.8), 4% SDS, 20% glycerol, 0.2% bromophenol blue, and 20% mercaptoethanol. The samples were heated for 15 min at 100 °C and loaded onto an SDS–PAGE gel. Proteins were transferred to a PVDF membrane, blocked with PBST buffer containing 5% bovine serum albumin (BSA)/0.1% Tween-20, and then probed at room temperature for 2 h with anti-NS1 polyclonal antibody (Gene Tex; GTX125990). An anti-GAPDH monoclonal antibody (Abcam; ab8245) was used as a loading control. A horseradish peroxidase (HRP) secondary antibody (ZSJQ-BIO; ZB2305/ZB2301) was used to detect primary antibody binding. Protein bands were visualized using SuperSignal West Femto’s highest sensitivity chemiluminescence substrate kit (Thermo Science, New York, NY, USA; 34096).

### 2.7. Pathogenicity Study in Mice

Five-week-old female C57BL/6N mice were purchased from Charles River (Beijing, China). All animal experiments were approved by the Ethical Committee of Beijing Institute of Microbiology and Epidemiology. To determine the MLD_50_ of influenza virus, four groups of mice (*n* = 5/group) were anesthetized with pentobarbital sodium (240 mg/kg body weight). One group was used as a negative control group (PBS-infected), another was used as a positive control group (CA07), and the remaining groups were infected with virus. Of these, one group was infected with XJ49-NS1mut virus (XJ49-NS1mut), and one group was infected with rXJ49 virus (rXJ49). The MLD50 of each virus was calculated using the method of Reed and Muench. Survival and body weight of each individual mouse was monitored daily for 15 days. Mice were sacrificed and scored dead when weight loss reached 25% of the baseline weight (i.e., the weight on the day of infection). Mice were sacrificed at 1, 3, and 6 dpi, and lungs were extracted and homogenized prior to measurement of virus titer. Virus titers were determined in a plaque assay in MDCK cells. Lungs were collected at 6 dpi and fixed with 4% neutral phosphate-buffered formalin. Fixed specimens were embedded in paraffin, sectioned, and stained with hematoxylin and eosin (H&E). For immunohistochemical staining, sections were probed with antibodies specific for the A/H1N1 HA protein or mouse IFN-β. Tissue sections were examined under a light microscope (PANNORAMIC DESK/MIDI/250/1000; 3DHISTECH (Budapest, Hungary)). Cytokine and chemokine expression in lung homogenates was measured using a ProcartaPlex Multiplex Immunoassay kit (Thermo Science, New York, NY, USA; MAN0016936).

### 2.8. Reporter System Assay

The 293T cells cultured in 24-well plates were transiently co-transfected with the reporter plasmid p125-luc encoding FF-Luc under the control of the IFN promoter, a plasmid pRL constitutively expressing Rluc under the control of the HSV–TK promoter, and the WT and mutant NS1 pHW2000-expressing plasmids (pHW2000-XJ49NS or pHW2000-NS1mut), using Lipofectamine 3000 reagent (ThermoFisher, New York, NY, USA; L3000-3000). The empty pHW2000 plasmid was used as a negative control, while poly (IC) was used as a positive control. At 24 h post-transfection, cells were harvested and lysed. Reporter activity was analyzed using a Dual-Luciferase Reporter Assay System kit (Promega, Beijing, China; E1910). All assays were performed independently and in triplicate.

### 2.9. qRT-PCR

Total RNA was extracted from cultured cells or lung homogenate using a PureLink viral RNA/DNA Mini kit (ThermoFisher, New York, NY, USA; 12280-050). Real-time PCR was performed using a One Step TB Green PrimeScript PLUS RT-PCR Kit (TaKaRa; RR096A) and a Light Cycler 480 II (Roche, Mannheim, Germany). The specific primers used to target human interferon and the amplification and quantitative real-time PCR (qRT-PCR) conditions can be provided upon request. Relative expression was calculated using the comparative ^ΔΔ^CT method. The results are expressed as the n-fold difference relative to expression of GADPH (2−^ΔΔ^CT).

### 2.10. Measurement of the IFN-β Levels

Culture supernatants were collected from A549 cells and assayed using the human interferon enzyme-linked immunosorbent assay kit (Gene-Protein Link, Beijing, China; P09H193).

### 2.11. Statistical Analysis

Data are presented as means ± SD of at least three independent experiments. Differences between two groups were evaluated using Student’s *t*-test, and differences between three or more groups were evaluated using analysis of variance (ANOVA). Statistical significance was set at *p* < 0.05. (*, *p* < 0.05; **, *p* < 0.01).

## 3. Results

### 3.1. The NS1 Dual R108K/G189D Mutant Virus Exhibited Similar Viral Replication Ability Compared with WT Strain

**Figure 1:**
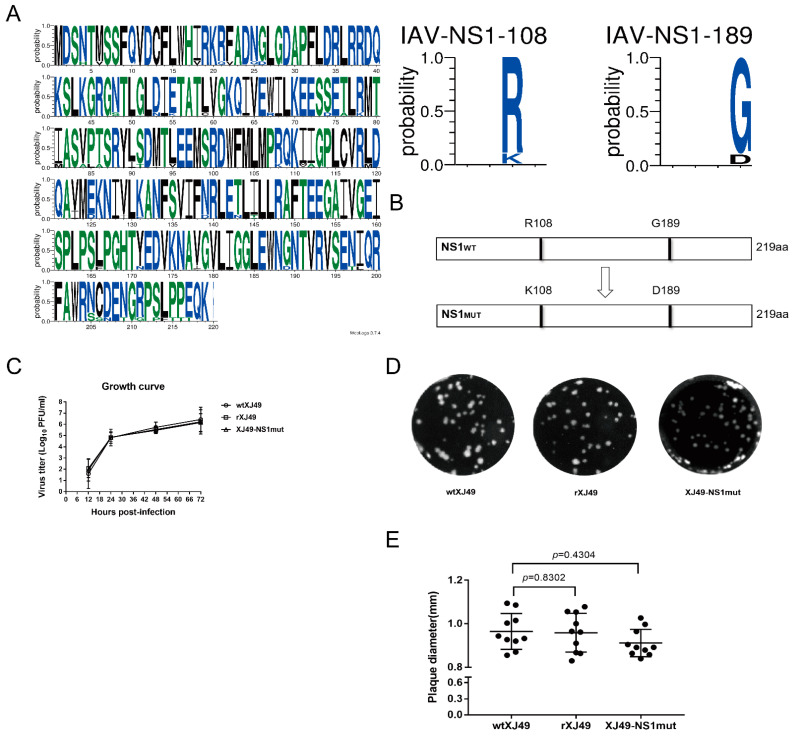
Characterization of the recombinant XJ49-NS1mut influenza virus. (**A**) Sequence logo describing sequence conservation at position 108 and 189 of the NS1 protein of the H1N1 virus (http://weblogo.threeplusone.com/create.cgi, accessed on 15 March 2020). (**B**) Schematic representation of the WT NS1 protein and the mutant NS1 protein harboring the R108K and G189D mutations. (**C**) Growth kinetics of the WT and recombinant XJ49-NS1mut viruses. MDCK cells were infected with wtXJ49, rXJ49, or XJ49-NS1mut at a MOI of 0.01. Supernatant was collected at 12, 24, 48, and 72 h post-infection, and the viral titer measured in a plaque assay. The mean yields and standard error of triplicate experiments are shown. (**D**) Plaque morphology and (**E**) average plaque size (*n* = 10 per virus). MDCK cells were infected with either WT or recombinant virus, and plaques were visualized by staining with crystal violet. There is no statistical difference (*p*: 0.8302, wtXJ49 vs. rXJ49; *p*: 0.3182, rXJ49 vs. XJ49-NS1mut; *p*: 0.4304, wtXJ49 vs. XJ49-NS1mut). Statistical analysis was performed using a two-tailed Student *t*-test (*p* < 0.05; *p* < 0.01).

To analyze whether the NS1 protein encoded by the human pH1N1 virus affects the growth and pathogenicity of the virus, the NS gene sequences of 2155 strains of H1N1 virus isolated since 2009 were downloaded and analyzed. Since the influenza A/H1N1 pandemic in 2009, NS1 proteins have evolved different proportions of R108 and K108, as well as of G189 and D189. Collective analysis of all available NS1 sequences of pdmH1N1 indicated that arginine (R) and glycine (G) are the predominant amino acids at positions 108 and 189, respectively (Figure 1A). The reverse genetics system of XJ49 was used to establish a seasonal 2009H1N1 influenza virus isolate (A/Urumqi/XJ49/2018(H1N1)). Using this reverse genetics system, dual R108K/G189D mutations were introduced into NS1 segments, and a recombinant mutant, rXJ49-NS1mut, was rescued successfully (Figure 1B). The growth kinetics of the rescued rXJ49 virus in MDCK cells were not significantly different from those of the parental WT strain wtXJ49 (Figure 1C). In MDCK cells, the rescued recombinant virus exhibited a plaque phenotype similar to that of the wtXJ49 virus (Figure 1D). In addition, there was no significant difference between the growth kinetics of XJ49-NS1mut and the parental rXJ49 virus (Figure 1C). The plaque diameters of wtXJ49, rXJ49, and XJ49-NS1mut were 0.964 ± 0.0261 mm, 0.958 ± 0.0280 mm, and 0.911 ± 0.0199 mm (Figure 1E), respectively. To examine the genetic stability of the mutant, rXJ49-NS1mut was passaged four times at a multiplicity of infection (MOI) of 0.01, and the titers of each generation were determined. Nucleic acids were extracted from the supernatants of the 3rd and 6th generations to determine the viral genome sequence and to identify whether substitutions had occurred. Sequencing of the NS fragment of rXJ49-NS1mut showed that the dual R108K/G189D mutation was stable over time.

### 3.2. In Vivo Characterization of the Recombinant pdmH1N1 Virus XJ49 Expression NS1 with Dual R108K/G189D Mutant

**Figure 2:**
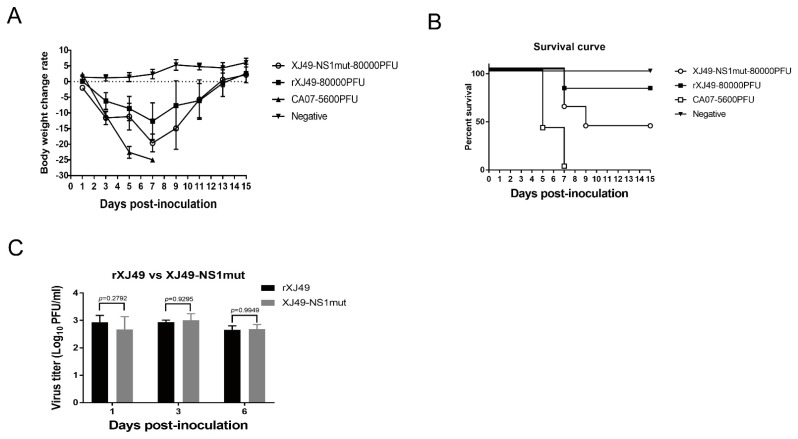
Virulence and replication of the WT rXJ49 influenza virus and the XJ49-NS1mut virus in the C57BL/6N mice. (**A**) Body weight changes in mice inoculated with 80000 PFU of rXJ49 or XJ49-NS1mut. (**B**) Survival curves of mice infected with 80000 PFU of rXJ49 or XJ49-NS1mut. PBS-infected mice were used as a negative control, while mice infected with 5600 PFU of A/California/07/2009 (H1N1) (GenBank Accession no. NC_026431-NC026438) were used as a positive control. Survival and weight loss were monitored daily. Mice were sacrificed and scored dead when they lost 25% of their baseline weight (measured on the day of infection). (**C**) Viral titers in the lungs of mice infected with the indicated viruses at 1, 3, and 6 dpi. Data are presented as the mean ± SD. Significance was calculated using one-way ANOVA with multiple comparison tests (*p* < 0.05; *p* < 0.01).

To test whether dual amino acid substitutions in the NS1 protein affect replication efficiency and pathogenicity of the 2009 pandemic A (H1N1) influenza viruses in vivo, The results showed that recombinant rXJ49-NS1mut was more pathogenic than WT rXJ49 virus (Figure 2A). The MLD_50_ of WT rXJ49 was 10^−2^ while that of rXJ49-NS1mut virus was 10^−1.3^ (Figure 2B). By contrast, there was no significant difference in the weight loss and viral load in the lungs of mice infected with these viruses (Figure 2A,C).

**Figure 3:**
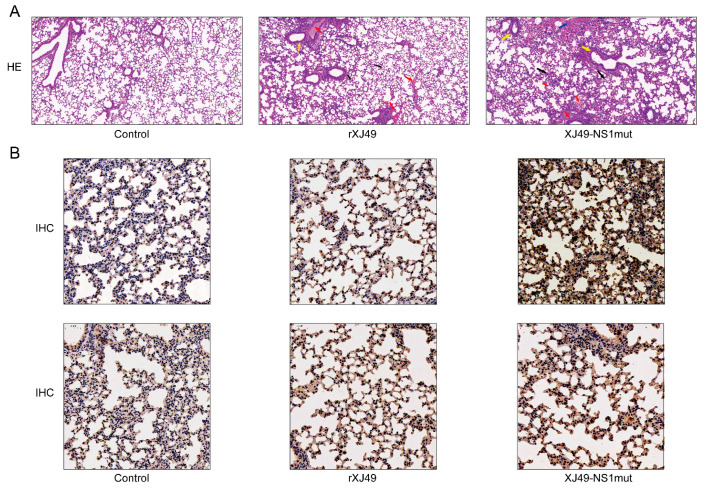
Pathological changes in the lungs of inoculated mice. C57BL/6N mice were infected intranasally with WT rXJ49 or rXJ49-NS1mut at a dose of 80,000 PFU. H&E (×100) and IHC (×50) analysis were performed on section of lung harvested from mice at 6 dpi. Solid arrows indicate inflammatory cell infiltration. (**A**) H&E-stained sections of lungs from mice infected with mock or the indicated viruses. The black arrows indicate large numbers of lymphocytes, neutrophils, and macrophages in the tissue. Yellow arrows indicate inflammatory cells infiltrating around local blood vessels to form a ring or vascular cuff. Red arrows indicate a small amount of bronchial epithelial cell necrosis and nuclear fragmentation locally. The blue arrow indicates local bleeding. (**B**, upper row) IAV HA protein-specific staining of lung sections. (**B**, lower row) IFN-β-specific staining of lung sections.

Pathological analysis of lung tissue from infected mice was performed at 6 dpi to further examine the pathogenic effects of rXJ49-NS1mut. Compared with those in the mock group (PBS-infected mice; Figure 3A, left), the lungs of WT rXJ49-infected mice showed mild to moderate inflammation, along with immune infiltration of the alveolar cavity and peribronchioles (Figure 3A, middle). By contrast, the lungs of XJ49-NS1mut-infected mice showed severe inflammatory cell infiltration and more local bleeding (Figure 3A, right). Immunohistochemical analyses revealed a large amount of viral antigen in the lungs of mice infected with influenza viruses. It also revealed greater numbers of virus-infected cells in the lungs of mice exposed to rXJ49-NS1mut than in those of mice exposed to WT rXJ49 virus (Figure 3B). These results suggest that the dual R108K/G189D mutation in the NS1 protein has little effect on viral replication but increases the pathogenicity of the XJ49 virus in mice.

### 3.3. The Dual R108K/G189D Mutant in NS1 Suppresses Expression of IFN-β

**Figure 4:**
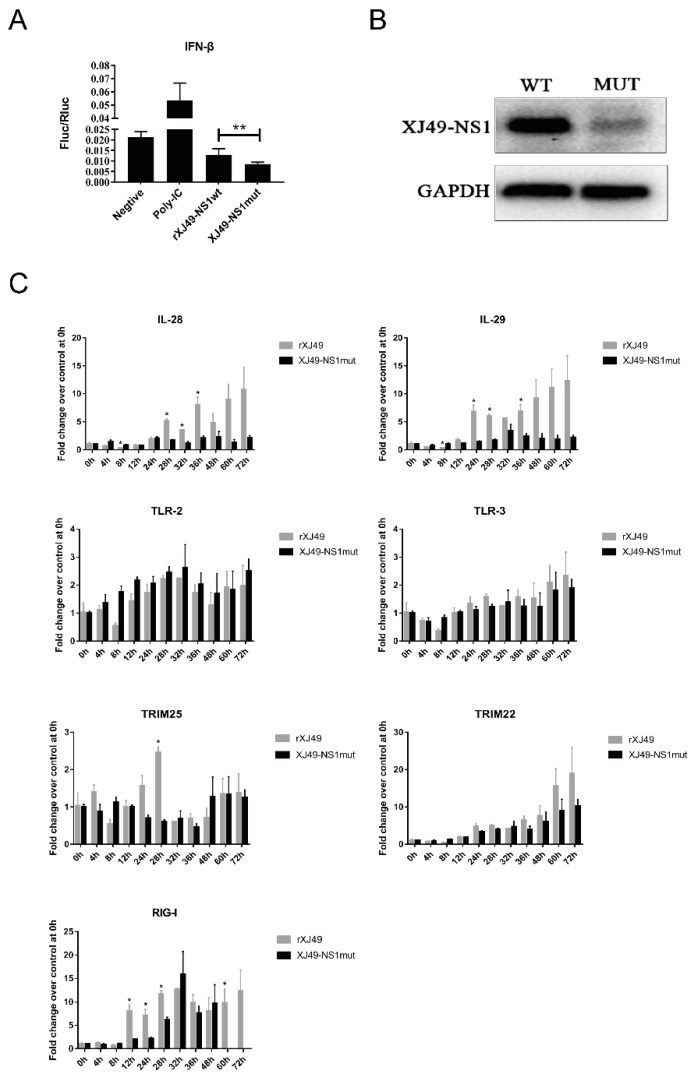
The H1N1 NS1 protein with the dual R108K/G189D mutation impairs host innate immune responses. (**A**) Ability of the mutant influenza NS1 protein to inhibit IFN-β promoter activation. Human 293T cells were transiently co-transfected with a reporter plasmid encoding FF–Luc under the control of the IFN promoter (p125-luc), a plasmid constitutively expressing Rluc under the control of the HSV–TK promoter (pRL), and the WT or mutant NS1 pW2000 expressing plasmids. Empty pHW2000 plasmid was used as a negative control, while poly (I:C) was used as a positive control (**, *p* < 0.001). (**B**) NS1 protein expression in transfected cells was evaluated by Western blotting with an antibody specific for the viral NS1 protein. GAPDH was used as a loading control. (**C**) Expression of cytokine mRNA in infected A549 cells. A549 cells were infected with rXJ49 and XJ49-NS1mut at a MOI of 5. The cells were collected at 0 h, 4 h, 12 h, 24 h, 28 h, 32 h, 36 h, 48 h, 60 h, and 72 h, and cytokine gene expression was quantified by qRT-PCR (*, *p* < 0.001).

To determine whether mutations R108K and G189D in the XJ49 NS1 protein affect IFN-β expression, we measured the promoter activity of IFN-β using a luciferase reporter gene assay. HEK293T cells were co-transfected with the IFN promoter–firefly luciferase reporter plasmid, p125-luc, the HSV–TK promoter–Renilla luciferase control plasmid pRL, and the NS1-expressing plasmid pHW2000-XJ49NS or pHW2000-NS1mut. Empty plasmid was used as a control, and poly (I:C) treatment was used as a positive control. After 24 h, the activity of FF–Luc was analyzed. As shown in Figure 4A, increased expression of IFN-β mRNA was observed in cells transfected with poly (I:C). Compared with the WT rXJ49 NS1 protein, the rXJ49-NS1mut protein harboring R108K/G189D inhibited activation of the IFN-β promotor. This suggests that the influenza virus NS1 protein inhibits host gene expression and that the R108K/G189D mutation increases this effect. In addition, expression of NS1 protein was measured by Western blotting (Figure 4B); the results showed that expression of mutant NS1 was significantly lower than that of WT NS1, supporting the results of the luciferase assay and suggesting that mutant NS1 inhibits host gene expression (Figure 4A,B). Thus, the R108K/G189D mutation increases the ability of NS1 to impair host innate immune responses.

### 3.4. Impact of the R108K/G189D Mutation on Expression of Genes Involved in Cytokine Responses In Vitro

To clarify how the XJ49-NS1mut virus affects host innate immune responses in vitro, A549 cells were infected with the indicated viruses, and expression of mRNA encoding key innate immune molecules (IL-28, IL-29, TLR-2, TLR-3, RIG-I, TRIM22, and TRIM25) was measured by qPCR (Figure 4C) after 24 h of infection. Expression of IL-28, IL-29, and TRIM22 mRNA induced by the parental strain increased from 24 h to 72 h post-infection. However, the NS1mut influenza virus significantly suppressed virus-induced innate immune responses; indeed, upregulation of IL-28, IL-29, and TRIM22 induced by WT NS1 was inhibited markedly. Importantly, IL-28 and IL-29 expression induced by the NS1mut virus was very low. Expression of TRIM25 by WT virus-infected cells increased markedly at 24 h post-infection, before falling rapidly at 28 h post-infection, whereas expression of TRIM25 in NS1mut-infected cells remained relatively stable during the course of infection. There was no difference in expression of TLR2 and TLR3 mRNA between cells infected with the WT or mutant viruses at 12 h post-infection. WT virus increased expression of mRNA encoding RIG-I, reaching a peak value at around 32 h post-infection; by contrast, RIG-I mRNA expression in NS1mut virus-infected cells rose slowly. These results suggest that the R108K/G189D mutation in NS1 inhibits expression of genes related to host innate immunity.

### 3.5. The XJ49-NS1mut Suppresses Host Innate Immune Response In Vivo

**Figure 5:**
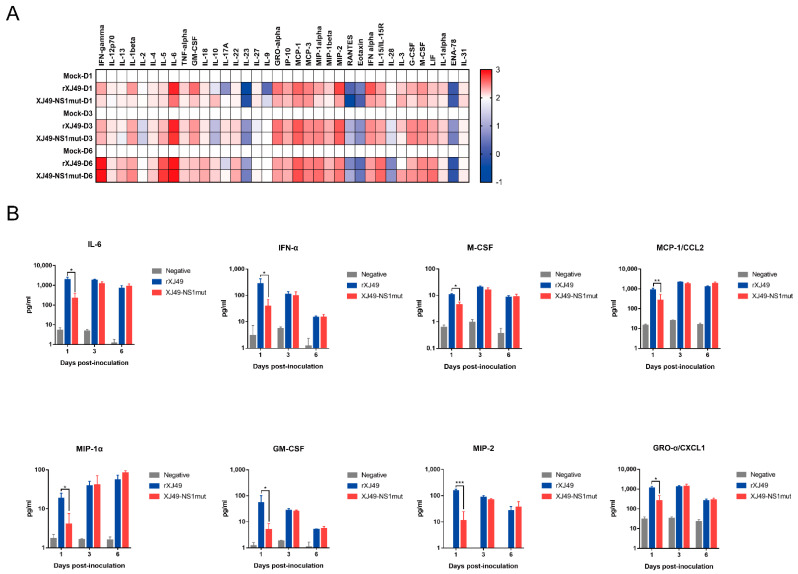
Differential expression of cytokines in the lung of mice infected with rXJ49 or XJ49-NS1mut. (**A**) Heat map showing differentially expression of cytokine-encoding genes in infected versus mock-infected lungs of C57BL/6N mice. Samples were analyzed using a 36-Plex mouse ProcartaPlex Panel kit. Expression of each cytokine is presented as log10-fold changes compared with the value in the mock group. (**B**) Expression of IL-6, GM-CSF, CXCL1, CCL2, MIP-1α, MIP-2, IFN-α, and M-CSF in C57BL/6N mice infected with rXJ49, rXJ49-NS1mut, or mock. Average of biological triplicates ± SD are shown. Significance was calculated using one-way ANOVA with multiple comparison tests (*, *p* < 0.05; **, *p* < 0.005; ***, *p* < 0.001).

Mice were infected with the WT rXJ49 virus or the rXJ49-NS1mut virus. At 6 dpi, mice were sacrificed, and lungs were extracted and homogenized. Cytokine and chemokine expression in lung homogenates was measured using the ProcartaPlex Multiplex Immunoassay. When a virus infects the host, it stimulates the immune system, which tries to remove the virus; here, we found that expression of cytokines involved in multiple pathways leading to IFN production were upregulated (Figure 5A); the exceptions were IL-2, IL-23, and RANTES. In response to viral infection, pulmonary epithelial cells and white blood cells produce chemokines and other proinflammatory cytokines, which play a role in limiting infection through cellular signaling. Among the selected cytokines or chemokines, IL-6, GM-CSF, CXCL1, CCL2, MIP-1α, MIP-2, IFN-α, and M-CSF (which are involved in acute inflammation and B cell maturation) were highly upregulated, although they exhibited transient inhibition at 1 day post-infection (Figure 5B). These results suggest that the R108K and G189D mutations in NS1 significantly inhibit expression of host-innate immunity-related proteins that function at the early stage of virus infection and that NS1 shutoff activity reported in previous studies is not the only mechanism of blocking antiviral responses in human hosts.

## 4. Discussion

The pathogenesis of IAV depends on the interaction between virus proteins and the host immune system. To replicate successfully in the host, IAVs have evolved to evade host immune response by encoding proteins such as NS1, which regulate host responses.

Previous studies show that the IAV NS1 protein binds to CPSF30, which suppresses expression of host genes encoding IFN and ISGs, thereby counteracting innate immune responses [17]. The NS1 protein of the 2009 pandemic H1N1 virus lost the ability to bind to CPSF30, so it does not block expression of these host genes [11,18]. Three amino acids (R108, E125, and G189) in the NS1 protein of the 2009 pandemic H1N1 virus are highly conserved, which is not the case for former seasonal influenza viruses and avian viruses. A previous study showed that reinstating R108K, E125D, and G189D rescued the ability to inhibit CPSF30 [11].

Here, we compared the sequences of 2155 strains of H1N1 virus identified since 2009 and found that, although some had R108K and G189D mutations in the NS1 protein, most retained R108 and G189. In 2018, we isolated a seasonal pandemic H1N1-origin influenza virus strain called A/Urumqi/XJ49/2018 from throat swabs of patients. We sequenced it and found that the NS1 protein contained the E125D mutation but retained R108 and G189. The isolate exhibited much lower morbidity in a mouse model than the early pandemic 2009 H1N1 isolate CA07. Thus, human H1N1 virus with triple or individual mutations at residues 108, 125, and 189 seemed not to be emerging constantly. Here, we used a H1N1 reverse genetics system to introduce mutations at sites 108 and 189 of the NS1 protein, hoping to obtain information about the molecular mechanism underlying how polymorphisms at these residues affect the interaction between NS1 and host factors. As expected, the rXJ49-NS1mut virus harboring the mutant NS1 protein replicated in MDCK cells in a manner similar to that of WT XJ49 virus but was more pathogenic in C57BL/6N mice. A previous study showed that a triple mutant NS1 virus based on the CA07 backbone showed less morbidity, whereas the mutant NS1 virus based on the XJ49 backbone exhibited higher morbidity. Nogales et al. revealed that inhibition of host gene expression depends on a strict balance between the NS1 and PA-X proteins. Compared with the pandemic CA07 virus, the seasonal XJ49 virus contained N204S, R221Q, L229S mutations in PA-X, which may be the reason for the different morbidity, even though it contained the same triple R108K, E125D, and G189D mutations [18].

Inhibition of interferon-induced cellular signals is very important for influenza infection, because production of cytokines such as type I interferon is the initial innate immune response that fights infection. The NS1 protein, which weakens the IFN response through a variety of mechanisms during influenza virus infection, is the most important IFN-antagonistic protein encoded by the virus [19]. The protein is highly expressed in the cytoplasm and nucleus of infected cells and can interact with different components involved in the IFN response [20]. During influenza virus infection, the host cell initiates a cascade of antiviral signals through pattern recognition receptor (PRR)-mediated recognition of pathogen-related molecular patterns (PAMPs). The main influenza virus PAMP is viral dsRNA with a triphosphate group at the 5’ end [21]. The NS1 protein can use its RNA binding region to bind competitively to PAMPs and inhibit activation of the RIG–I signal pathway, thereby inhibiting transcription and translation of the IFN-β promoter. Here, we used a double luciferase assay to examine the regulatory effect of the NS1 protein on the IFN promoter; we found that WT NS1 did not affect IFN expression, whereas NS1 harboring R108K/G189D suppressed it.

The TRIM family is a superfamily with Ring-finger E3 ubiquitin ligase activity, which has more than 70 members in humans. TRIM family members are critical regulators of innate immune responses against microbial pathogens, including viruses [22]. During influenza virus infection, NS1 inhibits oligomerization of TRIM25 by interacting with the E3 ubiquitin ligase domain to prevent ubiquitination of RIG-I K63, thereby blocking formation of the RIG-I-MAVS signal complex and effectively inhibiting production of IFN [9]. Early studies show that CPSF30 recognizes the polyadenylation signal at the 3’ end of host mRNA during transcription, cleaves host RNA, and binds poly A polymerase to increase the tail of poly A; thus, NS1 binding to CPSF30 can inhibit polyadenosine acidification at the 3’ end of mRNA and block mRNA production in the nucleus, thereby inhibiting host cell gene expression [23]. Here, we infected A549 cells with two viruses and found that expression of mRNA encoding TRIM25 and RIG-I in cells infected with XJ49-NS1mut was significantly lower than that in cells infected with rXJ49. Trim22 is a typical TRIM family protein that restricts replication of influenza viruses by targeting IAV nucleoproteins for degradation [24]. Published studies reveal that recognition of viral DNA/RNA triggers type I interferon production dependent on IRF3 activation. Secreted IFN-α/β bind to IFNAR to activate the Stat1–Stat2 heterodimer and induce expression of TRIM22, thereby protecting the host against diverse viral infections. The mutant NS1 protein may inhibit expression of TRIM25 and TRIM22, block the RIG–I pathway, and inhibit expression of IFN. In addition, the levels of IL-28 and IL-29 in cells infected with rXJ49-NS1mut decreased significantly, indicating that the R108K/G189D mutation in the NS1 protein could indeed inhibit host immunity during the early stage of virus infection by suppressing activation of the JAK–Stat pathway.

It seems that different cytokine waves appear as infection progresses. The first cytokines produced in the lungs after IAV infection are chemokines CCL5/RANTES, CCL2/MCP-1, and CXCL8/IL-8, as well as the main antiviral cytokines IFN-α, IFN-β, and IFN-γ. When the virus reaches the lower respiratory tract, it can infect activated alveolar macrophages and secrete higher levels of proinflammatory and antiviral cytokines [9]. In the lung homogenates of infected mice, we detected representative cytokines; indeed, expression of these cytokines in the lungs of mice infected with rXJ49-NS1mut and WT rXJ49 virus were significantly upregulated, especially those involved in inflammation (CCL2/MCP-1, CCL3/MIP-1α, CCL4/MIP-1β, and CCL7/MCP-3). Interestingly, we analyzed the pathology of mouse lungs and found that the pathological damage caused by rXJ49-NS1mut virus was more serious than that caused by WT rXJ49. It can be speculated that the R108K and G189D mutations in the NS1 protein enhance the innate immune response of the influenza virus against the host, resulting in the weakening of the host antiviral response, thus achieving the purpose of immune escape.
